# The scientific article and the good science

**DOI:** 10.5935/1678-9741.20150064

**Published:** 2015

**Authors:** Domingo M. Braile

**Affiliations:** 1 Editor-in-Chief - BJCVS/RBCCV

The scientific paper is one of the most visible areas of science. It is disseminating his ideas, new techniques and discoveries that the scientist, whether he is doctor, physician, biologist, engineer etc., plays his role in the expansion of knowledge and the consequent benefit to society and may break paradigms. This process is not simple, as we know, going through a series of bottlenecks ranging from consulting the relevant literature, research, data compilation, writing, journal's choice, where it passes through the sieve of the reviewers, final approval by the Editor and finally adjustments, in minor or major ways, until its release/publication.

Currently, the requirements are higher, either because postgraduate school to which the author is connected requires publishing studies in journals with higher impact factor (IF), depending on the CAPES criteria (Qualis Capes)^[1]^, in the case of Brazil, or the need for the article is written in English so it can be read, and eventually cited by the greatest number of readers.

In choosing a journal until the speed of dissemination of knowledge on the Internet, it is important, since today's novelty turns into something "outdated" tomorrow.

Aware of this new scenario, scientific journals have sought to refine their acceptance criteria and publishing articles. The Scielo has encouraged the journals that are part of its regular basis to take action accordingly. This creates a side effect, which is the increasing rejection of studies, either in submission or during the review process. In journals from various fields, not only among the best known, the rate of rejected manuscripts exceeds 90%^[2]^. In The Lancet, for example, it is 95%^[3]^. In journals restricted to a specific area such as the Brazilian Journal of Cardiovascular Surgery (BJCVS), the percentage is somewhat lower, but has been increasing over the years.

The BJCVS, aware that despite the new additions in recent years it still has a long way to go, has not measured efforts to walk "*pari passu*" with the best-known journals. Our rejection rate is rising gradually. In 2013, of 186 studies received, 61 were rejected (32.79%). In 2014, we received 162 manuscripts and 70 were rejected (43.21%). The average time between the submission of the study and the decision to approve or reject fell 147 days in 2013 to 86 days in the last year (Table 1).

**Table 1:** Number of manuscripts received, approved and rejected by the Brazilian Journal of Cardiovascular Surgery in 2013 and 2014*

	Item	Total 2014	Total 2013
1	Articles Sent	162	186
2	Articles Approved	65	104
3	Articles Refused	70	61
4	Time between submission and approval (days)	96	147

* The difference between the total number and sum of approved and disapproved articles justified by the fact that not all articles received in a given year are published in the same year of submission, as in the case in question.

Source: GN1 Sistemas e Publicações.

This shows our commitment to rigor and good science. Reviewers, also Brazilians overwhelmingly (Table 2), have been instructed to be strict and in some cases the study, when it is appropriate and does not have methodological errors, can incorporate adaptations suggested by the editor and reviewers, to be formatted according to the journal's standards and stand ready to be published in hard copy and/or electronic form.

**Table 2:** Countries of origin of authors and reviewers of manuscripts submitted to Brazilian Journal of Cardiovascular Surgery in 2013 and 2014.

	Country	Authors 2014	Authors 2013	Reviewers 2014	Reviewers 2013
1	Brazil	104	139	120	123
2	Turkey	9	12	0	0
3	China	6	4	0	0
4	Colombia	2	1	0	0
5	Portugal	1	0	1	1
6	Germany	1	0	0	0
7	Greece	1	2	0	0
8	Italy	1	0	0	0
9	Serbia and Montenegro	1	0	0	0
10	Venezuela	1	0	0	0
11	United States	0	2	2	2

Source: GN1 Sistemas e Publicações.

Seeking the rapid spread of the item after the approval, pending the publication of a new issue of the journal the manuscript is available "ahead of print" already with the DOI (Digital Object Identifier) allowing its citation in the literature.

Despite all our efforts to internationalize the BJCVS, it is still an eminently Brazilian journal. In 2013, of the total number of items received, 161 were from Brazil, followed by 14 from Turkey, 4 from China, 2 from the United States, 2 from Greece, 1 from Argentina, 1 from Colombia and 1 from the Netherlands. In 2014, 120 were from Brazil, 22 from China, 11 from Turkey, 2 from Colombia, 2 from Greece and 1 from Germany, 1 from Italy, 1 from Portugal, 1 from Serbia and 1 from Venezuela (Table 3).

**Table 3:** Countries of origin of the manuscripts submitted to Brazilian Journal of Cardiovascular Surgery in 2013 and 2014.

	Country	2014	2013
1	Brazil	120	161
2	China	22	4
3	Turkey	11	14
4	Colombia	2	1
5	Greece	2	2
6	Germany	1	0
7	Italy	1	0
8	Portugal	1	0
9	Serbia and Montenegro	1	0
10	Venezuela	1	0
11	Argentina	0	1
12	Netherlands	0	1
13	United States	0	2

Source: GN1 Sistemas e Publicações.

Due to the largest number of studies received, Brazil was the country with the largest number of rejected manuscripts (Table 4).

**Table 4:** Countries of origin of the manuscripts rejected by the Brazilian Journal of Cardiovascular Surgery in 2013 and 2014.

	Country	Without Referee 2014	With Referee 2014	Without Referee 2013	With Referee 2013
1	Brazil	25	27	18	31
2	Turkey	5	0	4	2
3	China	4	7	1	2
4	Germany	1	0	0	0
5	Argentina	0	0	0	1
6	Colombia	0	1	0	0
7	Greece	0	0	1	0
8	Netherlands	0	0	0	1

Source: GN1 Sistemas e Publicações.

With the adoption of English as the official language of the journal, six issues a year and a greater increase in disclosure, we hope to receive more international quality studies, and also from Brazil.

Without being redundant, I explain the reason to emphasize that the studies should be of quality and international. I see this as the only way to see our IF increase because the chance of readers of BJCVS to cite our journal in other publications that also are in the Thomson database and the Scopus increases in parallel to these parameters.

## Redalyc

Aiming to expand our visibility, after various requirements and a long time in another database, the Redalyc admitted us (http://www.redalyc.org/). Based in Mexico, Redalyc has nearly 1,000 open access journals, allowing the dissemination of scientific information.

## 30 years

As we reported in previous editions, we are preparing, together with the Board of the Brazilian Society of Cardiovascular Surgery (BSCVS), several activities to commemorate the 30^th^ anniversary of the BJCVS. In programming of the 43^rd^ Brazilian Congress of Cardiovascular Surgery, which will take place from 7 to 9 April 2016, in Fortaleza, there will be a module in the programming dedicated to our journal, where we discuss topics of interests of authors, reviewers and professionals of health field in general, who are interested in scientific production. We will also pay tribute to our partners, who over these nearly three decades spared no efforts so that the BJCVS could be what it is today.

We trust that even facing many obstacles, never interrupting the circulation, the perspective is positive for us to continue our successful track record. The journal is available on multiple platforms, is present in social networks and always attentive to new technology and, of course, always trying to improve its content.

## CME

The following items are available for the testing of Continuing Medical Education (CME) in this issue: "*Post-cardiotomy ECMO in pediatric and congenital heart surgery: impact of team training and equipment in the results*" (page 409); "*Relationship between pre-extubation positive end-expiratory pressure and oxygenation after coronary artery bypass grafting*" (page 443); "*Evaluation of the influence of pulmonary hypertension in ultra-fast-track anesthesia technique in adult patients undergoing cardiac surgery*" (page 449); "*Effects of intraoperative diltiazem infusion on flow changes in arterial and venous grafts in coronary artery bypass graft surgery*" (page 459). I emphasize that the CME is a valuable tool for learning and updating of knowledge and is worth 0.5 points in the BJSVC proof of title. We are open to suggestions and criticisms to improve the system.

My warmest regards,

Domingo Braile - BJCVS/RBCCV
